# JCDSA: a joint covariate detection tool for survival analysis on tumor expression profiles

**DOI:** 10.1186/s12859-018-2213-3

**Published:** 2018-05-29

**Authors:** Yiming Wu, Yanan Liu, Yueming Wang, Yan Shi, Xudong Zhao

**Affiliations:** 1 College of Information and Computer Engineering, Northeast Forestry University, No.26 Hexing Road, Harbin, 150001 China; 2 College of Foreign Languages, Northeast Forestry University, No.26 Hexing Road, Harbin, 150001 China

**Keywords:** Feature selection, Expression profiles, Survival analysis, Prognosis, Cancer

## Abstract

**Background:**

Survival analysis on tumor expression profiles has always been a key issue for subsequent biological experimental validation. It is crucial how to select features which closely correspond to survival time. Furthermore, it is important how to select features which best discriminate between low-risk and high-risk group of patients. Common features derived from the two aspects may provide variable candidates for prognosis of cancer.

**Results:**

Based on the provided two-step feature selection strategy, we develop a joint covariate detection tool for survival analysis on tumor expression profiles. Significant features, which are not only consistent with survival time but also associated with the categories of patients with different survival risks, are chosen. Using the miRNA expression data (Level 3) of 548 patients with glioblastoma multiforme (GBM) as an example, miRNA candidates for prognosis of cancer are selected. The reliability of selected miRNAs using this tool is demonstrated by 100 simulations. Furthermore, It is discovered that significant covariates are not directly composed of individually significant variables.

**Conclusions:**

Joint covariate detection provides a viewpoint for selecting variables which are not individually but jointly significant. Besides, it helps to select features which are not only consistent with survival time but also associated with prognosis risk. The software is available at http://bio-nefu.com/resource/jcdsa.

## Background

Due to the limited effectiveness of current clinical diagnoses, expression profiles are utilized for informing variables, which are not only associated with the categories of patients with different survival risks but also consistent with survival time [1]. Commonly, Cox proportional hazards regression analysis is used to seek relevant variables considering the continuity of the patients’ survival outcomes with right censoring [2]. As to small sample data with high dimension, Cox proportional hazards regression has to be combined with methods using dimension reduction or shrinkage such as partial least squares [3] and principal component analysis [4]. However, these approaches only provide a combination of variables. Besides, tree-structured survival analysis [5], random survival forests [6] and that associated with hazards regression [7] are proposed for selection of features associated with survival outcomes. Anyway, these top-down strategies provide so many variable candidates that the real features which may reveal the possible molecular cause of different survival risks are inevitably submerged.

In contrast, univariable hazards regression analyses have been placed firmly in the mainstream. Bottom-up strategies with different constraints such as least-angle regression [8] and sparse kernel [9] are utilized for providing variables associated with survival time. To the best of our knowledge, we are the first to present joint covariate detection [1] that combines significant variables consistent with survival time and associated with the categories of patients. Other than individually significant variables, we concentrate on bottom-up enumeration of feature tuples, each component of which is either individually significant or not. This thought is inspired by Integrative Hypothesis Testing [10], which is used for selecting features differentially expressed between different groups of patients. Unlike Integrative Hypothesis Testing, joint covariate detection is faced with continuous survival time other than labels representing different categories of patients.

In this paper, we further divide the provided feature selection into two steps, i.e., selection of variables associated with survival outcomes and further feature selection for discrimination between patients with different survival risks. In addition, we develop a joint covariate detection tool for survival analysis on tumor expression profiles (i.e. JCDSA), which helps to conveniently select significant features either on a cluster or a workstation, even on a personal computer. Matlab R2012b and Python 3 are utilized as the development platform. miRNA expression data (Level 3) of 548 patients with GBM downloaded from TCGA (http://cancergenome.nih.gov) and the simulated data are considered to be the examples. Compared with the prevailing method named as random survival forests (i.e. RSF), JCDSA shows better experimental results, which demonstrates the effectiveness of our method.

## Implementation

**Fig. 1:**
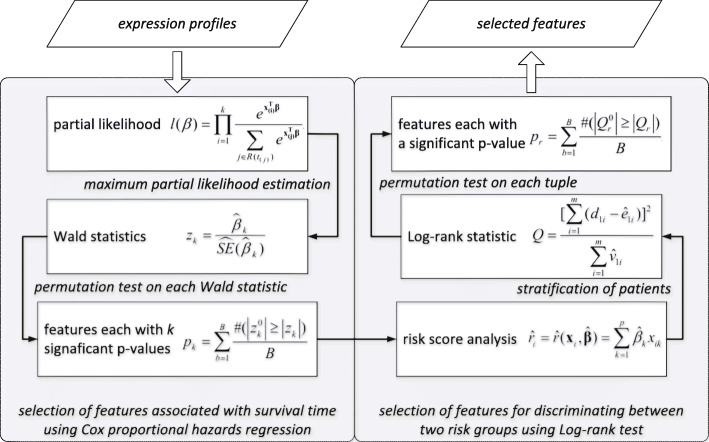
A schematic diagram to elucidate joint covariate detection

In order to elucidate joint covariate detection in brief, a schematic diagram is illustrated in Fig. 1 (Notations: **x**_(*i*)_ and **β** denote the expression levels of sample *i* and the regression coefficients of the detected variables, respectively. The summation in the denominator is over all subjects in the risk set at ordered survival time *t*_(*i*)_, denoted by *R*(*t*_(*i*)_). $z_{k}^{0}$ denotes a null statistics by a random rearrangement of survival outcomes. The estimator of the expected number of deaths in high-risk group is denoted by $\hat {e}_{1i}$, expressed as $\hat {e}_{1i}$ = $\frac {n_{1i}d_{i}}{n_{i}}$, where n _*i*_ and d _*i*_ represent the number at risk and of deaths at the observation of ordered survival time *t*_(*i*)_, *n*_1*i*_ denotes the number at risk in high-risk group. The estimator of the variance of *d*_1*i*_ on the hypergeometric distribution is defined as $\hat {v}_{1i}$ = $\frac {n_{1i}n_{0i}d_{i}(n_{i} - d_{i})}{n_{i}^{2}(n_{i} - 1)}$, where n _0*i*_ denotes the number at risk in low-risk group. Q$_{r}^{0}$ denotes a null statistics by a random rearrangement of survival outcomes). Input data is considered as expression profiles with survival time and censoring states of patients. Output data refers to selected features. Joint covariate detection corresponds to two-step feature selection, i.e., selection of features associated with survival outcomes and selection of features for discriminating between two risk groups.

### Features associated with survival outcomes

We first consider to select features associated with survival time. A bottom-up enumeration on *k*-tuple with *k* variables is made. As to each *k*-tuple, Cox proportional hazards regression analysis [2] is introduced. By making the maximum partial likelihood estimation on the partial likelihood function, we obtain *k* estimated regression coefficients on which Wald statistics are made. Furthermore, a permutation test is made on each Wald statistic. The *k*-tuple with each component corresponding to a significant *p* value is regarded as a candidate feature associated with survival outcomes. More details can be seen in [1].

### Features for discriminating between two risk groups

We then intend to select features for discriminating between low-risk and high-risk group of patients, which conforms to doctors’ daily decision making process. As to each patient, a risk score which is the linear portion of the expression values using the Cox regression coefficients is calculated. A preassigned risk score is utilized as a cut-off value for stratification between high-risk and low-risk group of patients. Log-rank test is made. Furthermore, a permutation test is presented on each tuple, which has been selected to be associated with survival outcomes. The *k*-tuple with a significant *p* value is regarded as a candidate feature for discriminating between two risk groups. More details can be also seen in [1].

### Brief overview of the software

Our software, which is implemented in Matlab R2012b or other later versions, can work on different computational platforms (e.g., a cluster, a workstation, even a personal computer). Therefore, it contains two parts, i.e., client and server. Selection of features associated with survival outcomes is accomplished by two Matlab m-files (i.e., ’/Client/S1_feature_selection.m’ and ’/Server/S1_feature_selection_on_server.m’). A further selection of features for stratification of patients is fulfilled by a Matlab m-file ’Client/S2_plot_draw.m’. If this program is implemented on a workstation or a personal computer, only the client part is needed. That is to say, users only need to concentrate on two GUIs (i.e., ’/Client/S1_feature_selection.m’ and ’Client/S2_plot_draw.m’) on the client part. Otherwise, the server part is also in demand. Data communications and environment configurations are actualized using Python 3. More details can be seen in the user’s guide on the website: http://bio-nefu.com/resource/jcdsa.

## Results

**Fig. 2:**
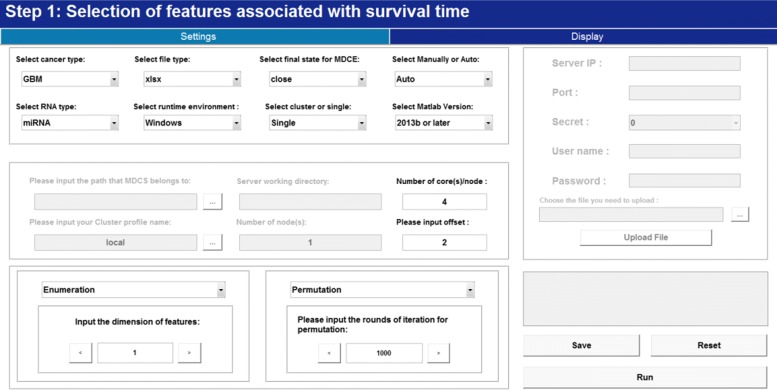
Selection of features associated with survival time

**Fig. 3:**
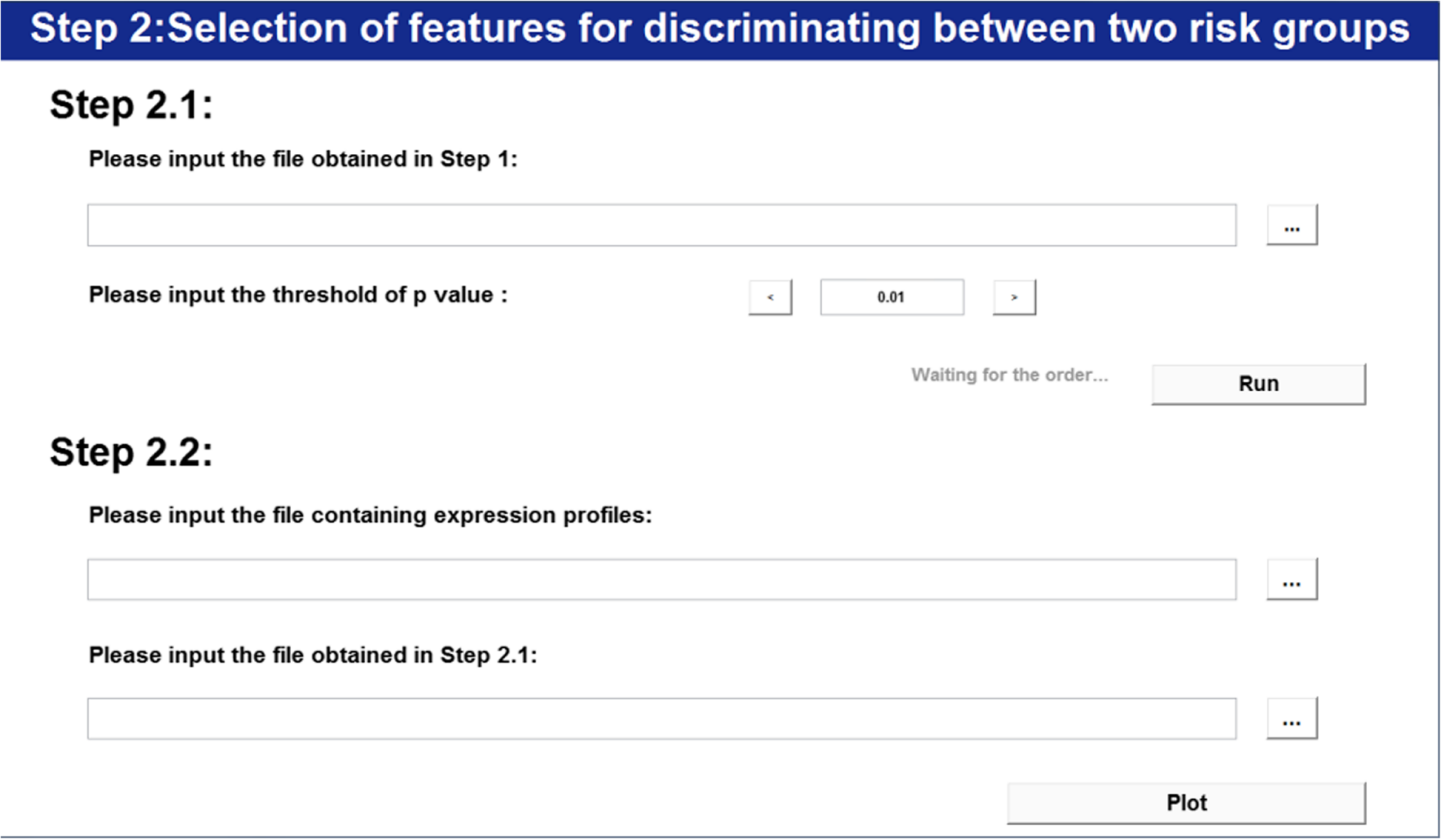
Selection of features for discriminating between two risk groups

According to the presented two-step feature selection strategy, we first consider selecting features associated with survival outcomes. Figure 2 illustrates this step. Cancer type can be selected or input by clicking the right side arrow if it is not supported in the type list. Other selections in the setting frame can be also made, details of which are listed in user’s guide. Before running at full speed, JCDSA estimates the finishing seconds which helps to make a further decision. After its completion, the result which records p value(s) of each *k*-tuple is stored in ’/Client/Data/S1’. Figure 3 further illustrates the step of selecting features associated with survival outcomes (i.e., Step 2.1). By setting the threshold of the p value corresponding to permutation test on Wald statistic, features associated with survival outcomes are selected.

Using the miRNA expression data (Level 3) of 548 patients with GBM as an example, individually significant miRNAs and significant miRNAs in pairs are listed in Tables 1 and 2, respectively. After making careful comparisons between Tables 1 and 2, we conclude that significant features in high dimension may not be composed of individually significant miRNAs. Taking the significant pair miR-10b and miR-222 as an example, miR-10b is not listed in Table 1, which shows that it is not individually significant. This phenomenon reveals the advantage of using joint covariate detection.

**Table 1:** Individually significant miRNAs using joint covariate detection (*p* <=0.001)

miRNA probe	*β*(Cox)	Z(Cox)	P(Cox)
hsa-miR-148a	0.192	4.607	<0.001
hsa-miR-17-3p	-0.308	-3.321	<0.001
hsa-miR-200a	0.465	3.563	<0.001
hsa-miR-20a	-0.177	-3.163	<0.001
hsa-miR-221	0.284	5.396	<0.001
hsa-miR-222	0.246	6.332	<0.001
hsa-miR-340	-0.468	-3.498	<0.001
hsa-miR-34a	0.182	4.287	<0.001

**Table 2:** Significant miRNAs in pairs using joint covariate detection (*p* <0.001)

miRNA probe	miRNA probe	*β*(Cox)	*β*(Cox)	Z(Cox)	Z(Cox)	P(Cox)	P(Cox)
hsa-miR-10b	hsa-miR-222	0.1412	0.3061	3.6472	7.1789	0.0004	<0.0001
hsa-miR-140	hsa-miR-148a	-0.2450	0.1956	-3.3193	4.7179	0.0004	<0.0001
hsa-miR-143	hsa-miR-34a	-0.2452	0.2326	-3.5230	5.2069	0.0004	<0.0001
hsa-miR-182	hsa-miR-204	-0.1186	0.1482	-3.4971	4.2846	0.0004	<0.0001
hsa-miR-340	hsa-miR-801	-0.7523	-0.2290	-4.7672	-4.0426	<0.0001	0.0002
hsa-miR-198	hsa-miR-671	0.6433	-0.6435	3.7746	-3.9295	0.0002	0.0002
hsa-miR-196a	hsa-miR-20a	0.2191	-0.2120	3.4284	-3.6662	0.0007	0.0002
hsa-miR-340	hsa-miR-452	-0.7811	-0.2872	-4.8128	-3.6202	<0.0001	0.0003
hsa-miR-196a	hsa-miR-20b	0.2159	-0.2582	3.3972	-3.6163	0.0008	0.0003
hsa-miR-196a	hsa-miR-340	0.2115	-0.5325	3.2889	-3.8183	0.0010	0.0003
hsa-miR-374	hsa-miR-671	-0.3845	-0.2770	-4.1883	-3.5837	0.0002	0.0004
hsa-miR-140	hsa-miR-801	-0.3620	-0.2002	-4.2702	-3.6236	<0.0001	0.0005
hsa-miR-340	hsa-miR-671	-0.7553	-0.2512	-4.6673	-3.4952	0.0002	0.0005
hsa-miR-340	hsa-miR-765	-0.7652	-0.2524	-4.6791	-3.4679	<0.0001	0.0006
hsa-miR-17-5p	hsa-miR-196a	-0.2635	0.2226	-3.8666	3.4765	<0.0001	0.0006
hsa-miR-222	hsa-miR-422b	0.2911	-0.3619	7.0607	-3.5045	<0.0001	0.0007
hsa-miR-140	hsa-miR-671	-0.3948	-0.2333	-4.2886	-3.3077	<0.0001	0.0007
hsa-miR-340	hsa-miR-370	-0.7885	-0.1201	-4.6899	-3.4386	<0.0001	0.0007
hsa-miR-374	hsa-miR-663	-0.3226	-0.2551	-3.9265	-3.4033	0.0002	0.0007
hsa-miR-190	hsa-miR-374	0.9479	-0.2649	3.4665	-3.5370	0.0004	0.0007
hsa-miR-148a	hsa-miR-30e-3p	0.2287	-0.3551	5.1831	-3.1949	<0.0001	0.0008
hsa-miR-374	hsa-miR-801	-0.2932	-0.1921	-3.7141	-3.4390	0.0005	0.0008
hsa-miR-374	hsa-miR-765	-0.3481	-0.2457	-3.9480	-3.2346	0.0002	0.0009
hsa-miR-30e-3p	hsa-miR-663	-0.4564	-0.2517	-3.4388	-3.2166	0.0005	0.0009
hsa-miR-181c	hsa-miR-675	-0.2618	-2.9279	-3.6755	-3.3646	0.0003	0.0010
hsa-miR-200b	hsa-miR-487b	0.4543	0.2424	4.0048	3.2972	0.0007	0.0010

**Fig. 4:**
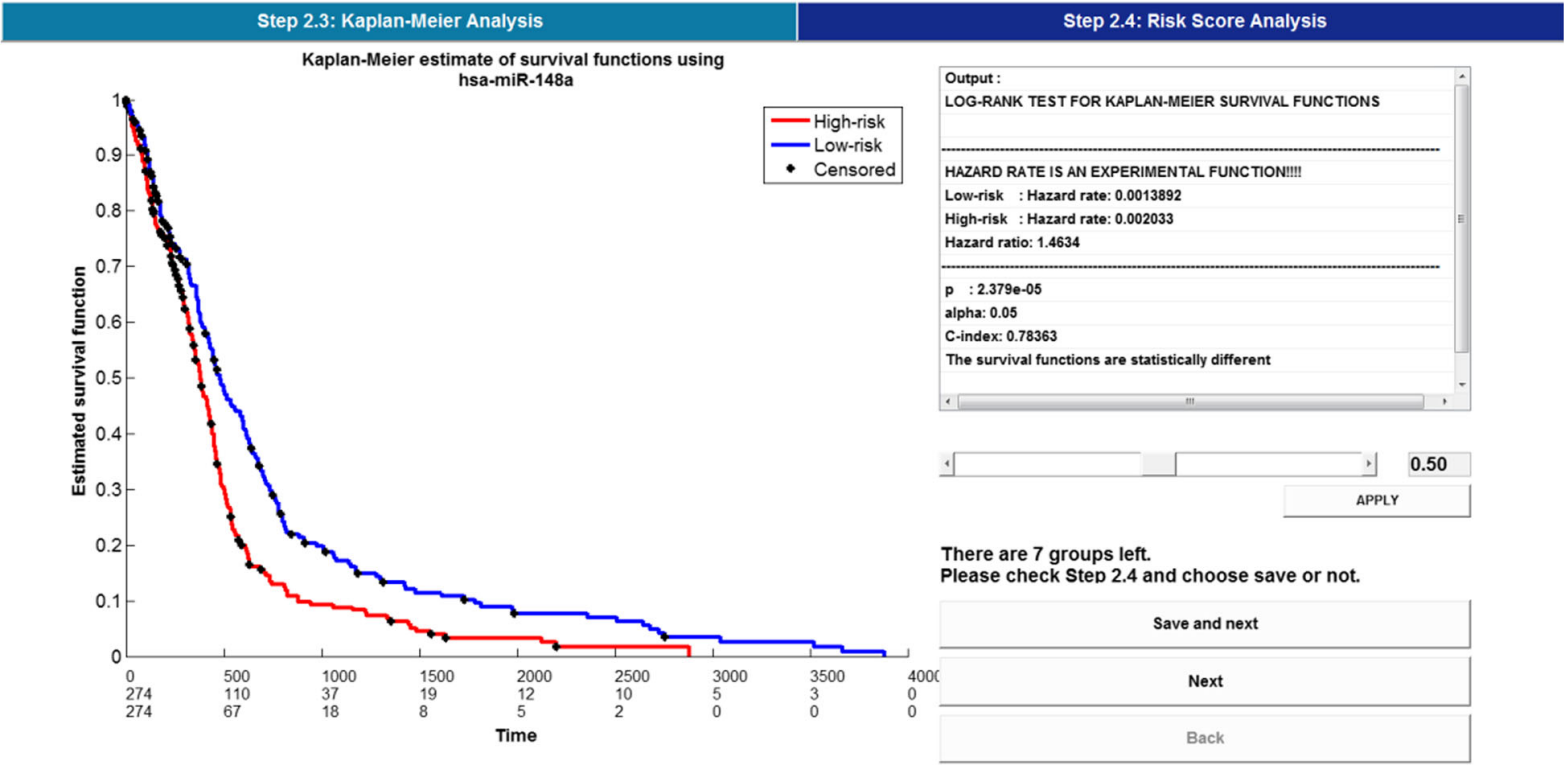
Kaplan-Meier analysis

**Fig. 5:**
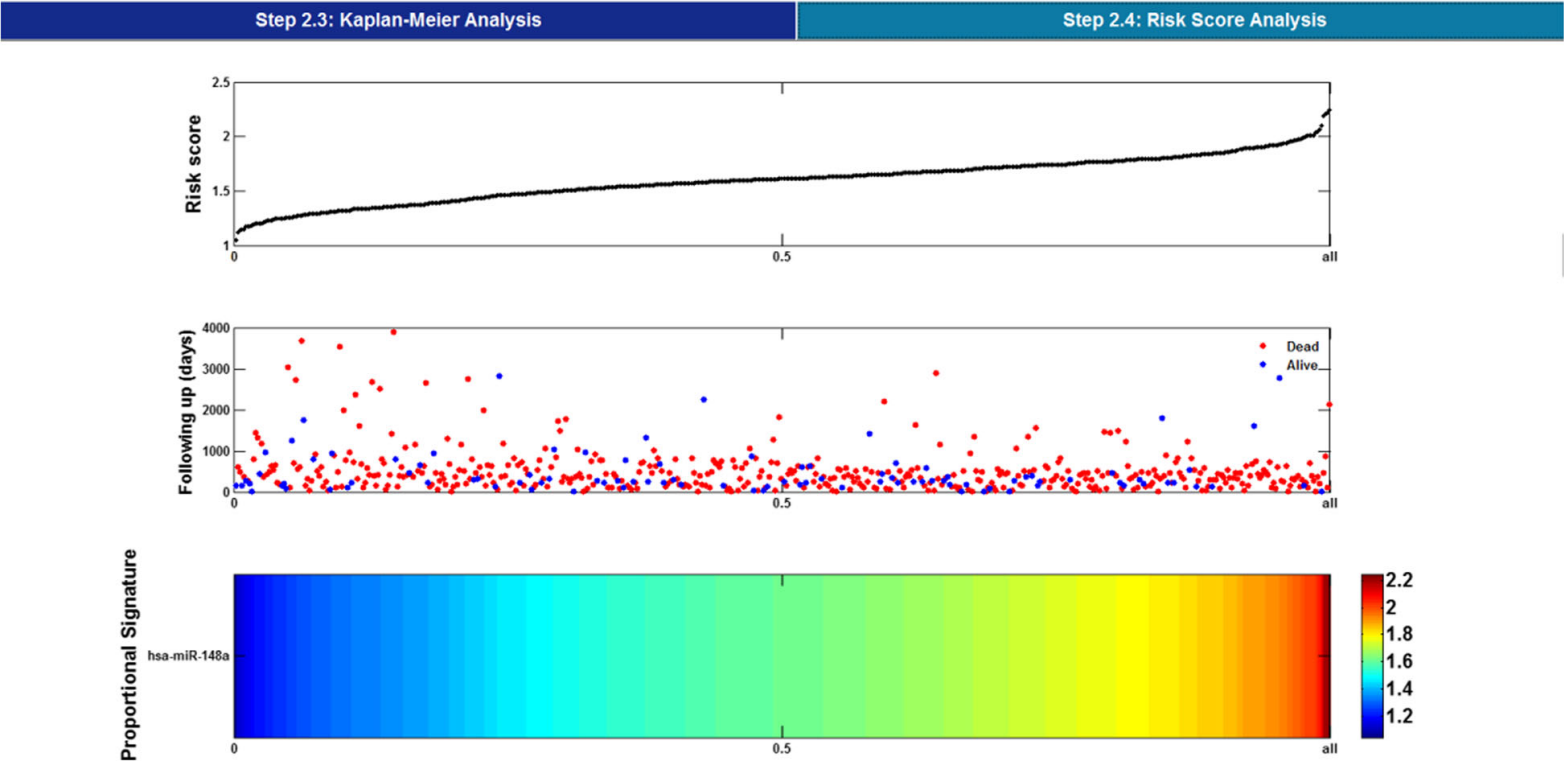
Risk score analysis

Together, Figs. 3, 4 and 5 illustrate the feature selection step for discriminating between two risk groups. In Fig. 3, after choosing the files that represent the original data and the result corresponding to significant features associated with survival time at Step 2.2, the software runs to Step 2.3 and Step 2.4.

As shown in Fig. 4, Kaplan-Meier analysis with parameters derived from log-rank test and Harrell’s concordance index is made for further selection of features, which helps to discriminate between high-risk and low-risk group of patients. Meanwhile, the result of risk score analysis is illustrated in Fig. 5. Correspondingly, results which refer to significant features are stored in ’Client/Data/S2/S2_3’ and ’Client/Data/S2/S2_4’, respectively.

In order to show the effectiveness of our method, we implemented the prevailing method named as random survival forests (i.e. RSF) on the miRNA expression data (Level 3) of 548 patients with GBM for comparison. 1000 binary survival trees were made, with each terminal node containing a minimum of *d*_0_=10 unique deaths. We made 1000 permutations on each variable, and obtained the variable importance (VIMP) for each variable. The result is listed in Table 3.

**Table 3:** Significant miRNAs using random survival forests (VIMP score >=0.001)

miRNA probe	VIMP score
hsa-miR-222	0.0103
hsa-miR-148a	0.0027
hsa-miR-30d	0.0012
hsa-miR-27a	0.0011
hsa-miR-422b	0.0011

After making careful comparisons between Tables 2 and 3, we find that miR-10b is still unimportant, as it is not listed in Table 3. This phenomenon reveals the advantage of using joint covariate detection other than RSF. In fact, the individually significant miR-222 keeps a *p*=0.0012 corresponding to log-rank test with 10000 rounds of permutation. As to significant pair (i.e., miR-222 and miR-10b), it keeps a *p*=0.0002 which corresponds to log-rank test with 10000 rounds of permutation. As to miR-10b, it keeps a *p*=0.285, which is individually insignificant.

We simulated data under 40 independent dimensions, from which we assigned two to be significant. That is, the survival time *S* is defined as *S*=*exp*(−**X***β*+*ε*), where **X** is the simulated gene expression matrix and *β*=[0.9,0.1,0.001,...,0.001]_40_ denotes the coefficient parameter. *ε*∼*N*(0,2). The sample size *n* is 50. The censoring states are generated, and yield 10 percent censoring for the simulated data.

The experimental results on simulated data are listed in Tables 4, 5 and 6, respectively. The significant pair closely associated with simulated survival outcomes are selected out, as shown in Table 5. In contrast, miRNA-alternative 2 which is in absence in Table 4 shows insignificant (*p*=0.939), and illustrates less important in Table 6. These results demonstrate the effectiveness of our method. The simulated data and full tables corresponding to Tables 4, 5 and 6 can be downloaded on the website: http://bio-nefu.com/resource/jcdsa.

**Table 4:** Individually significant miRNAs using joint covariate detection on the simulated data (*p* <=0.05)

miRNA probe	*β*(Cox)	Z(Cox)	P(Cox)
miRNA-alternative 1	4.739	5.929	<0.001
miRNA-null 33	-0.3583	-1.9486	0.023

**Table 5:** Significant miRNAs in pairs using joint covariate detection on the simulated data (*p* <=0.001)

miRNA probe	miRNA probe	*β*(Cox)	*β*(Cox)	Z(Cox)	Z(Cox)	P(Cox)	P(Cox)
miRNA-alternative 1	miRNA-alternative 2	7.6975	0.8455	5.1236	3.6895	<0.001	<0.001

**Table 6:** Significant miRNAs using random survival forests on the simulated data (VIMP score >=0.001)

miRNA probe	VIMP score
miRNA-alternative 1	0.1887
miRNA-null 32	0.0016
miRNA-alternative 2	0.0013
miRNA-null 10	0.0013

**Fig. 6:**
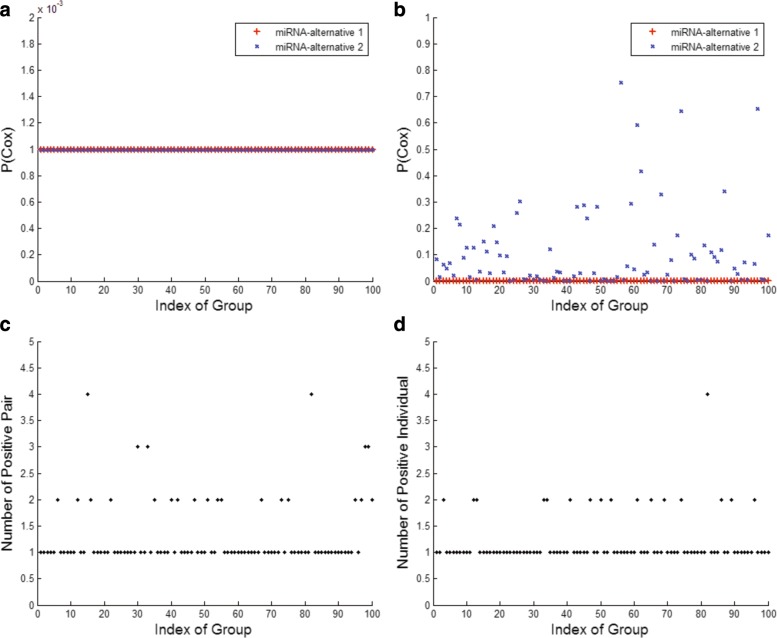
Simulation results. **a** p values of the significant pair through 100 times of simulation. **b** p values of the significant individual through 100 times of simulation. **c** The number of positive pairs through 100 times of simulation. **d** The number of positive individuals through 100 times of simulation

In order to show that selected variables are improbable false positive or false negative ones, we repeated the simulations above for 100 times with an enlarged sample size (*n*=500). The experimental results are illustrated in Fig. 6. Figure 6a denotes the p values (*p*<1*e*−3) of the significant pair through 100 times of simulation. However, miRNA-alternative 2 individually shows less important, as illustrated in Fig. 6b. Comparisons between Fig. 6a and b indicate that the significant features are probably not composed of individually significant uni-variables. Figure 6c and d report the number of positive pairs and individuals through 100 times of simulation, respectively. No false negative results are discovered. In Fig. 6c, the maximum number of false positive pair is three, which indicates a small probability of false positive pair 0.0038 (i.e., $3/C_{40}^{2}$). As to Fig. 6d, the maximum number of false positive individual is also three; yet, the probability of false positive individual is 0.075 (i.e., 3/40).

## Discussion

There are several states needed to be discussed. First, it is the significant multi-variable other than combinations of individually significant uni-variables that contributes to selection of features not only consistent with survival outcomes but also associated with stratification of patients under different survival risks. This fact has been demonstrated by our experimental results in this paper. Second, components of each significant multi-variable may keep a low correlation. This phenomenon has been discovered when experiments on the simulated data were made. Further evidence is still needed. Third, the correction for multiple hypothesis testing is absent, considering the computational cost of calculating FDR, q value, the adjusted p values, etc. on each pair or each high-dimension tuple of variables. However, simulations are made, which demonstrate the effectiveness of our method.

## Conclusion

Our joint covariate detection for survival analysis provides a new viewpoint for selecting variable candidates which are not individually but jointly significant. Following a two-step variable selection strategy, we propose a software (i.e., JCDSA) in order to help users to select features which are not only consistent with survival time but also associated with prognosis risk. JCDSA can be adapted for many categories of cancer. Users can easily operate it and conveniently obtain the experimental results for subsequent biological experimental validation.

## Availability and requirements

**Project name**: JCDSA **Project home page**: http://bio-nefu.com/resource/jcdsa**Operating system(s)**: Linux, Windows **Programming language**: Matlab (≥R2012b), Python (≥ 3.0) **License**: GPL (≥2) **Any restrictions to use by non-academics**: none

## Abbreviations

GUI: Graphical user interface
GBM: Glioblastoma multiforme
JCDSA: Joint covariate detection for survival analysis
TCGA: The Cancer Genome Atlas
